# 1624. SARS-CoV-2 Maternal Vaccination Induces More Efficient Placental Antibody Transfer to Newborns than SARS-CoV-2 Infection During Pregnancy

**DOI:** 10.1093/ofid/ofad500.1459

**Published:** 2023-11-27

**Authors:** Leire Pérez Latorre, Shira H Cohen, Paula Rodriguez-Molino, Hannah Kim, Mariah Eisner, Traci Pifer, Manish Rijal, Zhaohui Xu, Maged M Costantine, Kara Rood, Mahmoud Abdelwahab, Osvaldo Reyes, Rodrigo DeAntonio, Xavier Saez-Llorens, Sara Mertz, Fang Ye, Desiree Jones, Anna Bartholomew, Mark E Peeples, Pablo J Sanchez, Asuncion Mejias, Octavio Ramilo

**Affiliations:** Center for Vaccines & Immunity at Abigail Wexner Research Institute at Nationwide Children's Hospital, Columbus, OH, USA, Madrid, Madrid, Spain; Nationwide Children's Hospital, Columbus, Ohio; Hospital La Paz Hospital, Madrid, Spain., Madrid, Madrid, Spain; Research Institute at Nationwide Children's Hospital, Cincinnati, Ohio; Biostatistics Resource at Nationwide Children's Hospital, Columbus, Ohio; Nationwide Children's Hospital, Columbus, Ohio; Nationwide Children's Hospital, Columbus, Ohio; Nationwide Children's Hospital, Columbus, Ohio; The Ohio State University Wexner Medical Center, Columbus, Ohio; The Ohio State University, Columbus, Ohio; The Ohio State university, Columbus, Ohio; Hospital Santo Tomás, Panama, Panama, Panama; CEVAXIN Centro de Vacunación e Investigación, Panama, Panama, Panama; Hospital del Niño Dr José Renán Esquivel, Panama, Panama, Panama; The Research Institute at Nationwide Children's Hospital, Columbus, Ohio; Nationwide Childrens Hospital, Columbus, Ohio; Nationwide Children's Hospital, Columbus, Ohio; Ohio State University, Columbus, Ohio; Center for Vaccines & Immunity at Abigail Wexner Research Institute at Nationwide Children's Hospital, Columbus, OH, USA, Columbus, Ohio; Nationwide Children's Hospital - The Ohio State University, Columbus, OH; St Jude Children's Researh Hospital, Memphis, Tennessee; St. Jude Children's Research Hospital, Memphis, Tennessee

## Abstract

**Background:**

Passive immunization via maternal antibodies (Ab) is a key mechanism for infant protection in early life. This study analyzed the landscape of passive immunization induced by SARS-CoV-2 infection and/or vaccination by comparing the magnitude and durability of the transferred Ab.

**Methods:**

Prospective multicenter observational cohort study of SARS-CoV2-infected and/or vaccinated pregnant women and their infants. We collected maternal and cord blood samples at delivery and neonatal/infant samples at delivery, and at 1, 2, 6 and 12 months (mo) of age. RBD and Spike IgG Ab titers were measured by ELISA.

**Results:**

215 mothers (infected=106, vaccinated=67, infected and vaccinated=42) and 174 infants (n=87, n=54, n=33, respectively) were enrolled. At birth RBD Ab titers median [IQR] of infants from vaccinated mothers [4.28 (3.48-4.80)] log_10_ ng/ml and from infected and vaccinated mothers [4.61 (4.27-4.93)] log_10_ ng/ml were higher than those from infected mothers [2.20 (0.10-3.30)] log_10_ ng/ml, p< 0.001. These differences were observed until 6 mo of age, when only 1/13 (7.7%) infant from infected mothers had detectable maternal Ab compared to 14/15 (93.3%) and 8/8 (100%) from the vaccinated, and infected and vaccinated groups, respectively. At 12 mo none of the infants born to infected mothers had detectable maternal Ab, while in the vaccinated group (n=11), 3 (27.3%) had persistent Ab, and 4 (36.4%) suspected infection. At 12 mo in the infected and vaccinated group (n=6) 3 had no Ab, and 3 had suspected infection. The median [IQR] placental Ab transfer ratio was higher in the vaccinated only group (2.94 [1.34-3.74]) than the infected only group (1.19 [0.33-2.52]) p< 0.01. Linear regression analyses demonstrated that the mean RBD Ab transfer ratio for infants from vaccinated vs infected women was 1.76 (95% CI=1.07-2.88, p=0.025) higher after adjusting for trimester of infection or vaccination. Similar results were observed with Spike Ab.

Demographic and clinical characteristics of the study population

**Figure:**
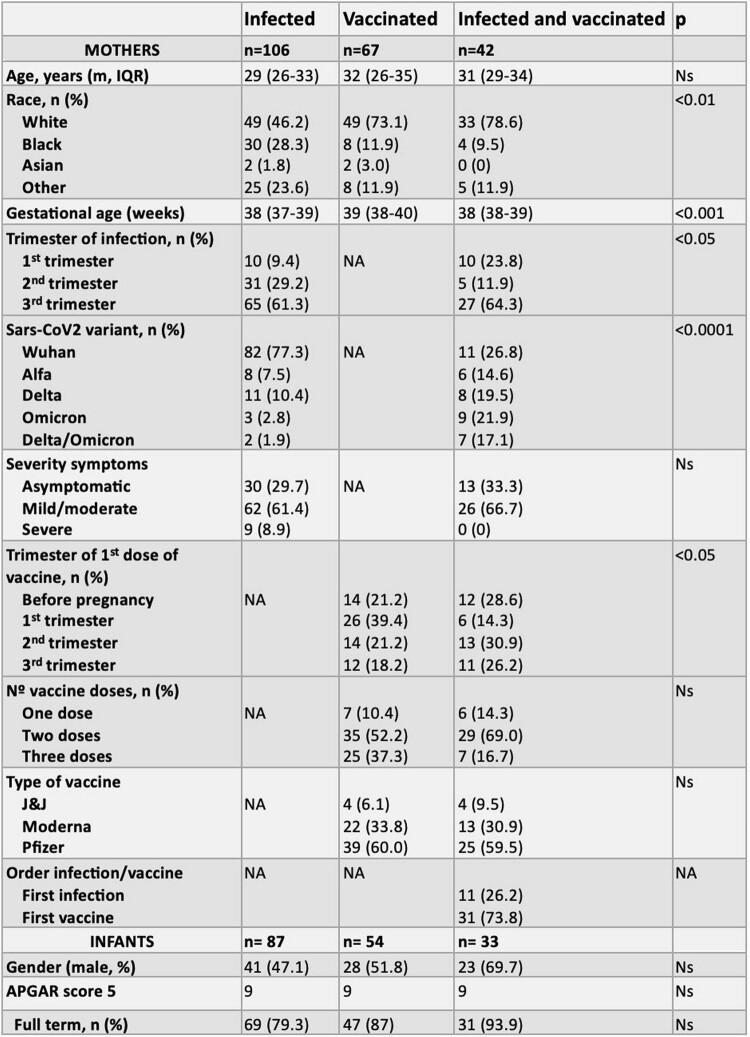

m; median. IQR; interquartile range. N/A; not applicable.

**Figure 1.**
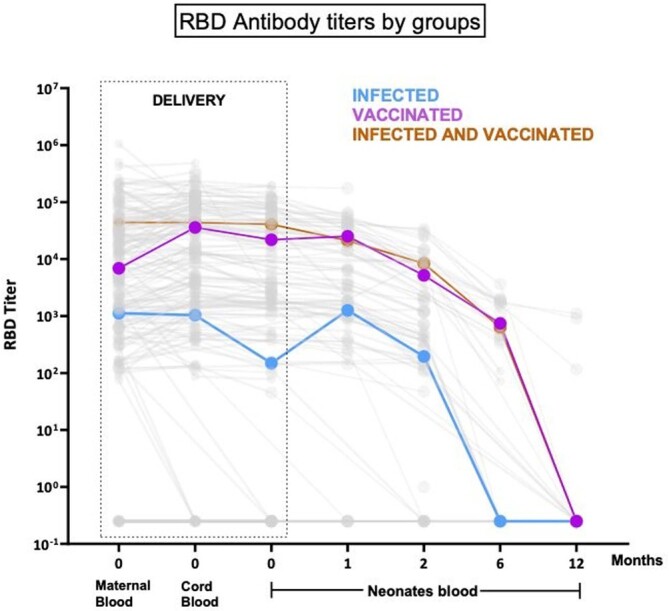
Comparison of RBD antibody titers in infected, vaccinated and infected and vaccinated groups at the time of delivery (from maternal blood, cord blood and neonatal blood) and at months 1, 2, 6 and 12 from neonatal/infant blood.

**Figure 2.**
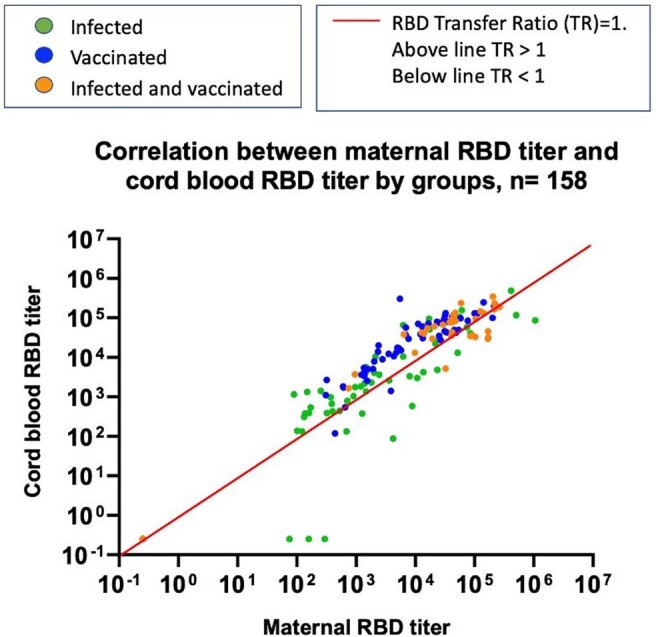
Correlation between maternal and neonatal RBD antibody titers in groups of infected, vaccinated and infected and vaccinated mothers. These correlations also represent the values of the antibody transfer ratios across the placenta. While the bisector (red line) represents the values for a transfer ratio (TR) = 1, all values above it would correspond with TR>1 and those below with TR < 1. Green dots represent the infected group, the blue dots the vaccinated group, and the orange dots the infected and vaccinated group.

**Conclusion:**

Infants from vaccinated mothers demonstrated maternal Ab up to 6 mo with higher titers than infants born from infected mothers. Antibodies generated by vaccination were transferred more efficiently than those generated by infection regardless of the trimester in which occurred.

**Disclosures:**

**Asuncion Mejias, MD, PhD, MsCS**, Astra-Zeneca: Advisor/Consultant|Merck: Grant/Research Support|Pfizer: Advisor/Consultant|Sanofi-Pasteur: Advisor/Consultant **Octavio Ramilo, MD**, AstraZeneca: Honoraria|Bill & Melinda Gates Foundation: Grant/Research Support|Janssen: Grant/Research Support|Merck: Advisor/Consultant|Merck: Grant/Research Support|Merck: Honoraria|NIH: Grant/Research Support|Pfizer: Advisor/Consultant|Pfizer: Honoraria|Sanofi: Advisor/Consultant|Sanofi: Honoraria

